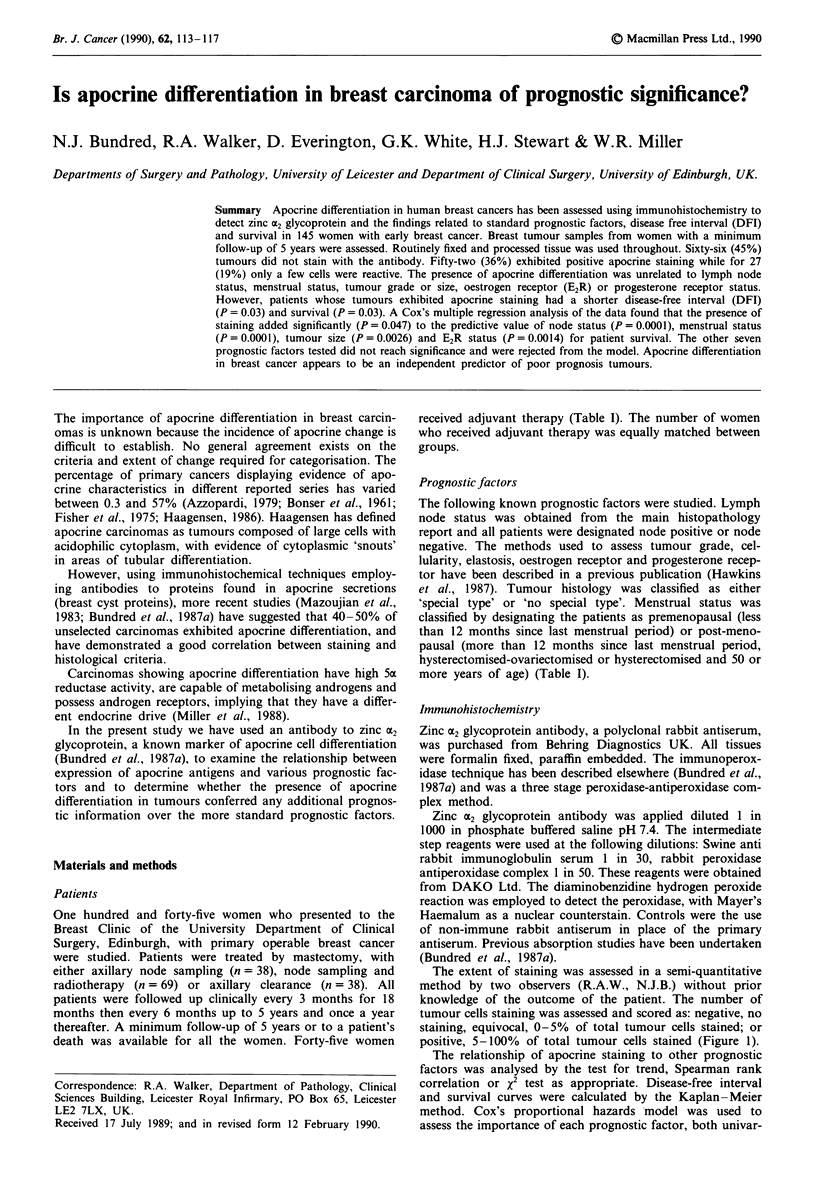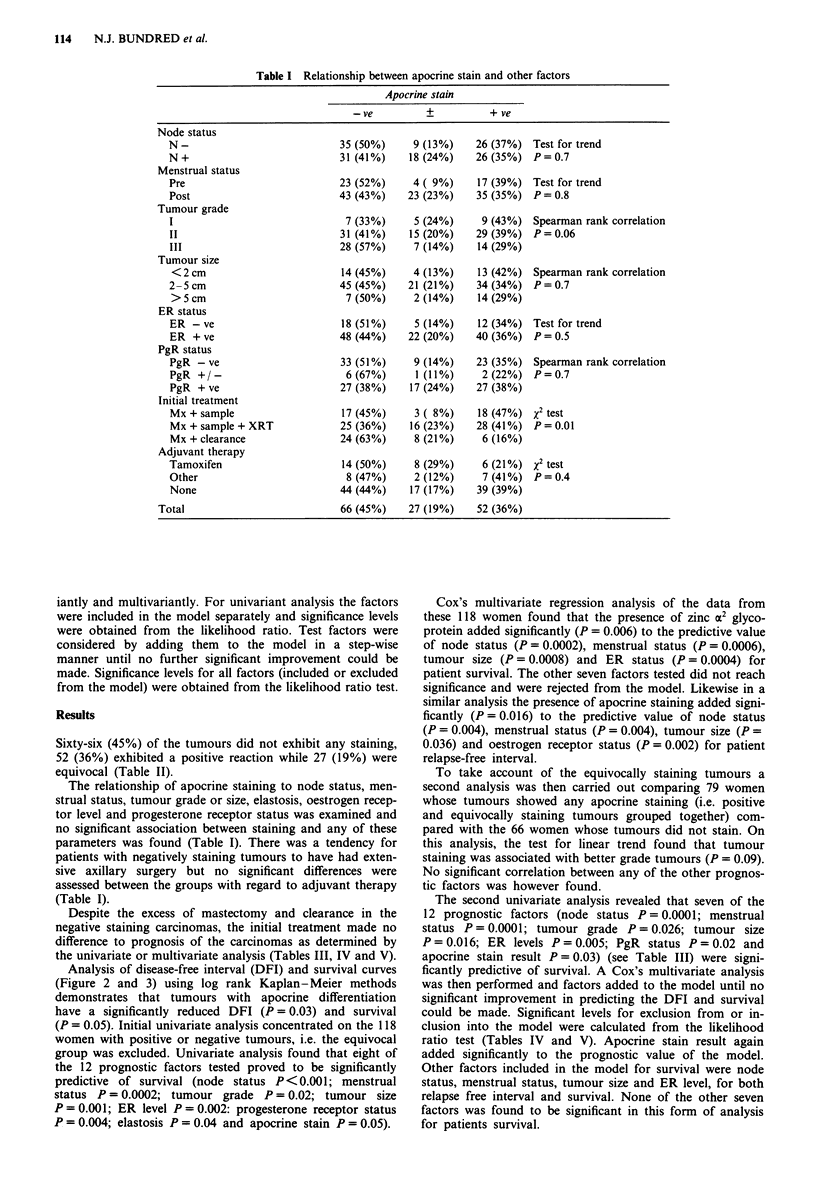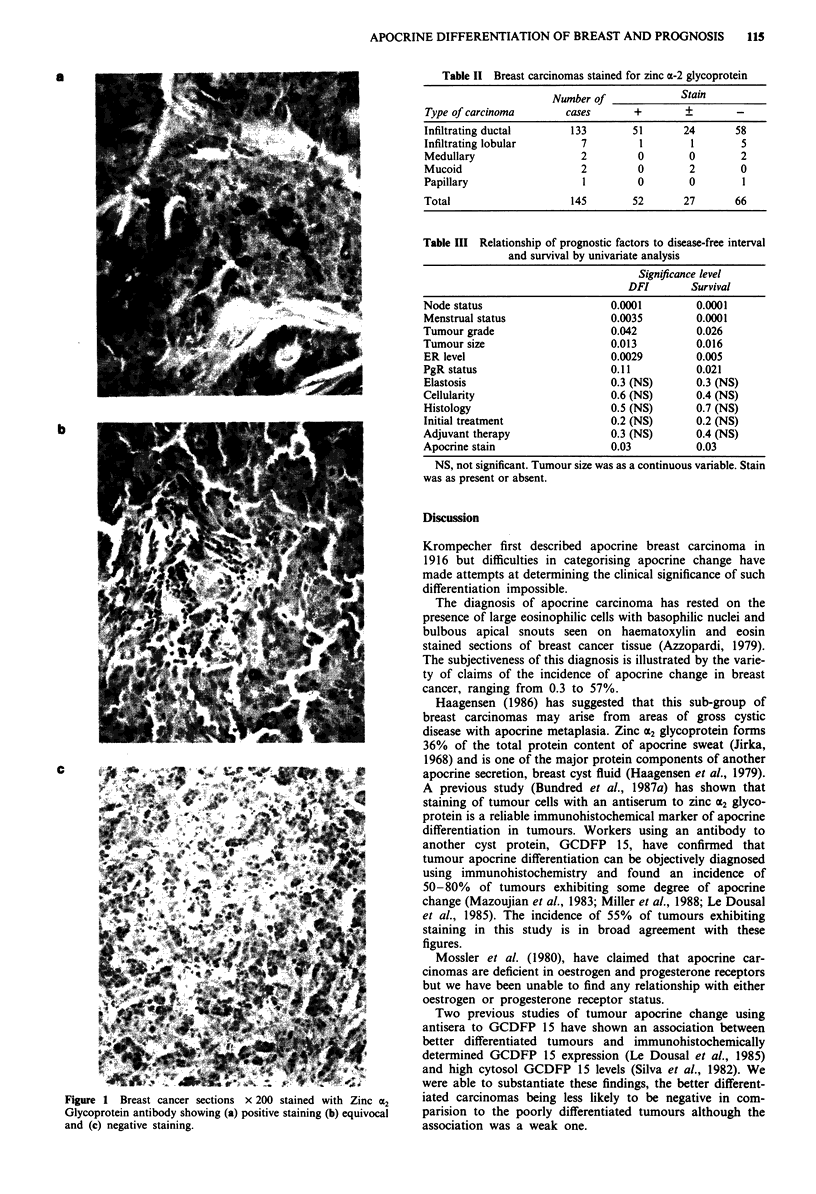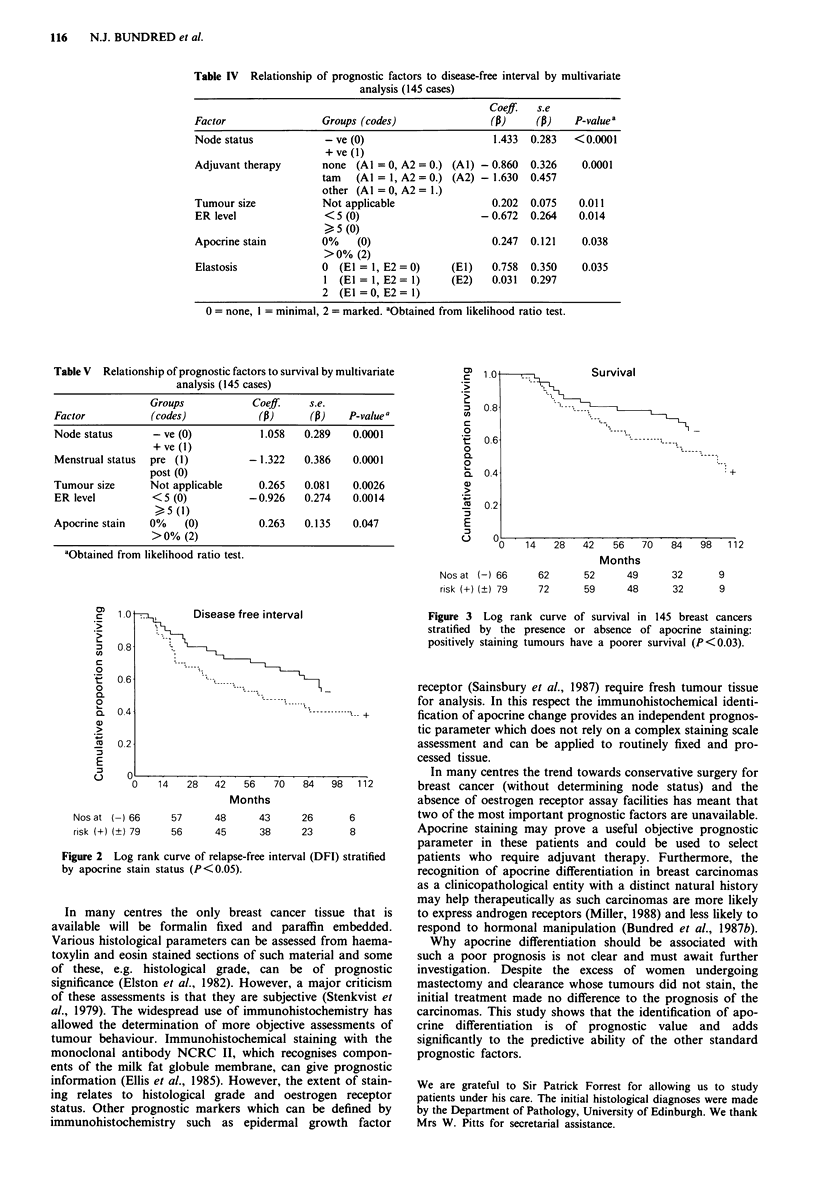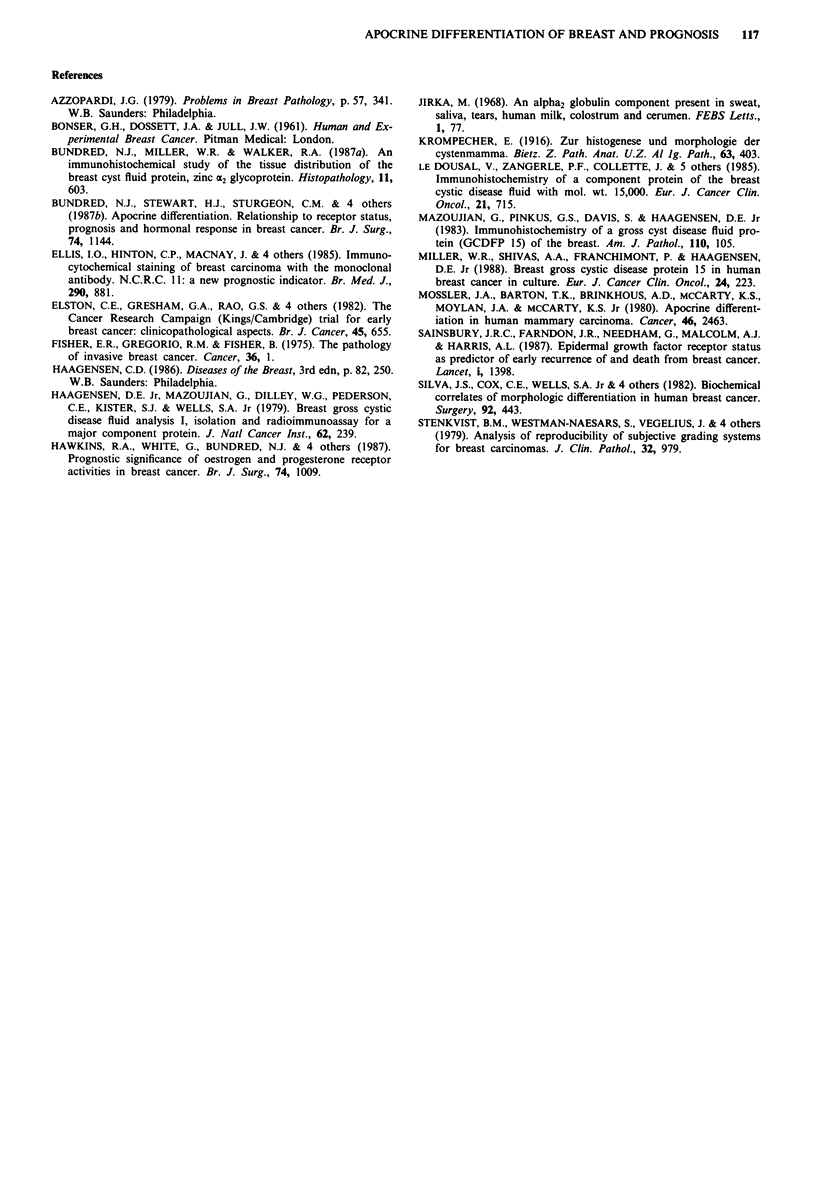# Is apocrine differentiation in breast carcinoma of prognostic significance?

**DOI:** 10.1038/bjc.1990.240

**Published:** 1990-07

**Authors:** N. J. Bundred, R. A. Walker, D. Everington, G. K. White, H. J. Stewart, W. R. Miller

**Affiliations:** Department of Surgery, University of Leicester, UK.

## Abstract

**Images:**


*Br. J. Cancer* (1990), **62**, 113–117
© Macmillan Press Ltd., 1990

### Is apocrine differentiation in breast carcinoma of prognostic significance?

N.J. Bundred, R.A. Walker, D. Everington, G.K. White, H.J. Stewart & W.R. Miller

*Departments of Surgery and Pathology, University of Leicester and Department of Clinical Surgery, University of Edinburgh, UK.*

**Summary** Apocrine differentiation in human breast cancers has been assessed using immunohistochemistry to detect zinc $\alpha_2$ glycoprotein and the findings related to standard prognostic factors, disease free interval (DFI) and survival in 145 women with early breast cancer. Breast tumour samples from women with a minimum follow-up of 5 years were assessed. Routinely fixed and processed tissue was used throughout. Sixty-six (45%) tumours did not stain with the antibody. Fifty-two (36%) exhibited positive apocrine staining while for 27 (19%) only a few cells were reactive. The presence of apocrine differentiation was unrelated to lymph node status, menstrual status, tumour grade or size, oestrogen receptor ($E_2R$) or progesterone receptor status. However, patients whose tumours exhibited apocrine staining had a shorter disease-free interval (DFI) ($P = 0.03$) and survival ($P = 0.03$). A Cox's multiple regression analysis of the data found that the presence of staining added significantly ($P = 0.047$) to the predictive value of node status ($P = 0.0001$), menstrual status ($P = 0.0001$), tumour size ($P = 0.0026$) and $E_2R$ status ($P = 0.0014$) for patient survival. The other seven prognostic factors tested did not reach significance and were rejected from the model. Apocrine differentiation in breast cancer appears to be an independent predictor of poor prognosis tumours.

The importance of apocrine differentiation in breast carcinomas is unknown because the incidence of apocrine change is difficult to establish. No general agreement exists on the criteria and extent of change required for categorisation. The percentage of primary cancers displaying evidence of apocrine characteristics in different reported series has varied between 0.3 and 57% (Azzopardi, 1979; Bonser *et al.*, 1961; Fisher *et al.*, 1975; Haagensen, 1986). Haagensen has defined apocrine carcinomas as tumours composed of large cells with acidophilic cytoplasm, with evidence of cytoplasmic 'snouts' in areas of tubular differentiation.

However, using immunohistochemical techniques employing antibodies to proteins found in apocrine secretions (breast cyst proteins), more recent studies (Mazoujian *et al.*, 1983; Bundred *et al.*, 1987*a*) have suggested that 40–50% of unselected carcinomas exhibited apocrine differentiation, and have demonstrated a good correlation between staining and histological criteria.

Carcinomas showing apocrine differentiation have high 5$\alpha$ reductase activity, are capable of metabolising androgens and possess androgen receptors, implying that they have a different endocrine drive (Miller *et al.*, 1988).

In the present study we have used an antibody to zinc $\alpha_2$ glycoprotein, a known marker of apocrine cell differentiation (Bundred *et al.*, 1987*a*), to examine the relationship between expression of apocrine antigens and various prognostic factors and to determine whether the presence of apocrine differentiation in tumours conferred any additional prognostic information over the more standard prognostic factors.

### Materials and methods

### *Patients*

One hundred and forty-five women who presented to the Breast Clinic of the University Department of Clinical Surgery, Edinburgh, with primary operable breast cancer were studied. Patients were treated by mastectomy, with either axillary node sampling ($n = 38$), node sampling and radiotherapy ($n = 69$) or axillary clearance ($n = 38$). All patients were followed up clinically every 3 months for 18 months then every 6 months up to 5 years and once a year thereafter. A minimum follow-up of 5 years or to a patient's death was available for all the women. Forty-five women received adjuvant therapy (Table I). The number of women who received adjuvant therapy was equally matched between groups.

### *Prognostic factors*

The following known prognostic factors were studied. Lymph node status was obtained from the main histopathology report and all patients were designated node positive or node negative. The methods used to assess tumour grade, cellularity, elastosis, oestrogen receptor and progesterone receptor have been described in a previous publication (Hawkins *et al.*, 1987). Tumour histology was classified as either 'special type' or 'no special type'. Menstrual status was classified by designating the patients as premenopausal (less than 12 months since last menstrual period) or post-menopausal (more than 12 months since last menstrual period, hysterectomised-ovariectomised or hysterectomised and 50 or more years of age) (Table I).

### *Immunohistochemistry*

Zinc $\alpha_2$ glycoprotein antibody, a polyclonal rabbit antiserum, was purchased from Behring Diagnostics UK. All tissues were formalin fixed, paraffin embedded. The immunoperoxidase technique has been described elsewhere (Bundred *et al.*, 1987*a*) and was a three stage peroxidase-antiperoxidase complex method.

Zinc $\alpha_2$ glycoprotein antibody was applied diluted 1 in 1000 in phosphate buffered saline pH 7.4. The intermediate step reagents were used at the following dilutions: Swine anti rabbit immunoglobulin serum 1 in 30, rabbit peroxidase antiperoxidase complex 1 in 50. These reagents were obtained from DAKO Ltd. The diaminobenzidine hydrogen peroxide reaction was employed to detect the peroxidase, with Mayer's Haemalum as a nuclear counterstain. Controls were the use of non-immune rabbit antiserum in place of the primary antiserum. Previous absorption studies have been undertaken (Bundred *et al.*, 1987*a*).

The extent of staining was assessed in a semi-quantitative method by two observers (R.A.W., N.J.B.) without prior knowledge of the outcome of the patient. The number of tumour cells staining was assessed and scored as: negative, no staining, equivocal, 0–5% of total tumour cells stained; or positive, 5–100% of total tumour cells stained (Figure 1).

The relationship of apocrine staining to other prognostic factors was analysed by the test for trend, Spearman rank correlation or $\chi^2$ test as appropriate. Disease-free interval and survival curves were calculated by the Kaplan–Meier method. Cox's proportional hazards model was used to assess the importance of each prognostic factor, both univar-

Correspondence: R.A. Walker, Department of Pathology, Clinical Sciences Building, Leicester Royal Infirmary, PO Box 65, Leicester LE2 7LX, UK.
Received 17 July 1989; and in revised form 12 February 1990.

**Table I** Relationship between apocrine stain and other factors

| | Apocrine stain | | | |
|---|---|---|---|---|
| | *− ve* | ± | *+ ve* | |
| Node status | | | | |
| N − | 35 (50%) | 9 (13%) | 26 (37%) | Test for trend |
| N + | 31 (41%) | 18 (24%) | 26 (35%) | $P = 0.7$ |
| Menstrual status | | | | |
| Pre | 23 (52%) | 4 ( 9%) | 17 (39%) | Test for trend |
| Post | 43 (43%) | 23 (23%) | 35 (35%) | $P = 0.8$ |
| Tumour grade | | | | |
| I | 7 (33%) | 5 (24%) | 9 (43%) | Spearman rank correlation |
| II | 31 (41%) | 15 (20%) | 29 (39%) | $P = 0.06$ |
| III | 28 (57%) | 7 (14%) | 14 (29%) | |
| Tumour size | | | | |
| <2 cm | 14 (45%) | 4 (13%) | 13 (42%) | Spearman rank correlation |
| 2–5 cm | 45 (45%) | 21 (21%) | 34 (34%) | $P = 0.7$ |
| >5 cm | 7 (50%) | 2 (14%) | 14 (29%) | |
| ER status | | | | |
| ER − ve | 18 (51%) | 5 (14%) | 12 (34%) | Test for trend |
| ER + ve | 48 (44%) | 22 (20%) | 40 (36%) | $P = 0.5$ |
| PgR status | | | | |
| PgR − ve | 33 (51%) | 9 (14%) | 23 (35%) | Spearman rank correlation |
| PgR +/− | 6 (67%) | 1 (11%) | 2 (22%) | $P = 0.7$ |
| PgR + ve | 27 (38%) | 17 (24%) | 27 (38%) | |
| Initial treatment | | | | |
| Mx + sample | 17 (45%) | 3 ( 8%) | 18 (47%) | $\chi^2$ test |
| Mx + sample + XRT | 25 (36%) | 16 (23%) | 28 (41%) | $P = 0.01$ |
| Mx + clearance | 24 (63%) | 8 (21%) | 6 (16%) | |
| Adjuvant therapy | | | | |
| Tamoxifen | 14 (50%) | 8 (29%) | 6 (21%) | $\chi^2$ test |
| Other | 8 (47%) | 2 (12%) | 7 (41%) | $P = 0.4$ |
| None | 44 (44%) | 17 (17%) | 39 (39%) | |
| Total | 66 (45%) | 27 (19%) | 52 (36%) | |

iantly and multivariantly. For univariant analysis the factors were included in the model separately and significance levels were obtained from the likelihood ratio. Test factors were considered by adding them to the model in a step-wise manner until no further significant improvement could be made. Significance levels for all factors (included or excluded from the model) were obtained from the likelihood ratio test.

### Results

Sixty-six (45%) of the tumours did not exhibit any staining, 52 (36%) exhibited a positive reaction while 27 (19%) were equivocal (Table II).

The relationship of apocrine staining to node status, menstrual status, tumour grade or size, elastosis, oestrogen receptor level and progesterone receptor status was examined and no significant association between staining and any of these parameters was found (Table I). There was a tendency for patients with negatively staining tumours to have had extensive axillary surgery but no significant differences were assessed between the groups with regard to adjuvant therapy (Table I).

Despite the excess of mastectomy and clearance in the negative staining carcinomas, the initial treatment made no difference to prognosis of the carcinomas as determined by the univariate or multivariate analysis (Tables III, IV and V).

Analysis of disease-free interval (DFI) and survival curves (Figure 2 and 3) using log rank Kaplan–Meier methods demonstrates that tumours with apocrine differentiation have a significantly reduced DFI ($P = 0.03$) and survival ($P = 0.05$). Initial univariate analysis concentrated on the 118 women with positive or negative tumours, i.e. the equivocal group was excluded. Univariate analysis found that eight of the 12 prognostic factors tested proved to be significantly predictive of survival (node status $P < 0.001$; menstrual status $P = 0.0002$; tumour grade $P = 0.02$; tumour size $P = 0.001$; ER level $P = 0.002$: progesterone receptor status $P = 0.004$; elastosis $P = 0.04$ and apocrine stain $P = 0.05$).

Cox's multivariate regression analysis of the data from these 118 women found that the presence of zinc $\alpha^2$ glycoprotein added significantly ($P = 0.006$) to the predictive value of node status ($P = 0.0002$), menstrual status ($P = 0.0006$), tumour size ($P = 0.0008$) and ER status ($P = 0.0004$) for patient survival. The other seven factors tested did not reach significance and were rejected from the model. Likewise in a similar analysis the presence of apocrine staining added significantly ($P = 0.016$) to the predictive value of node status ($P = 0.004$), menstrual status ($P = 0.004$), tumour size ($P = 0.036$) and oestrogen receptor status ($P = 0.002$) for patient relapse-free interval.

To take account of the equivocally staining tumours a second analysis was then carried out comparing 79 women whose tumours showed any apocrine staining (i.e. positive and equivocally staining tumours grouped together) compared with the 66 women whose tumours did not stain. On this analysis, the test for linear trend found that tumour staining was associated with better grade tumours ($P = 0.09$). No significant correlation between any of the other prognostic factors was however found.

The second univariate analysis revealed that seven of the 12 prognostic factors (node status $P = 0.0001$; menstrual status $P = 0.0001$; tumour grade $P = 0.026$; tumour size $P = 0.016$; ER levels $P = 0.005$; PgR status $P = 0.02$ and apocrine stain result $P = 0.03$) (see Table III) were significantly predictive of survival. A Cox's multivariate analysis was then performed and factors added to the model until no significant improvement in predicting the DFI and survival could be made. Significant levels for exclusion from or inclusion into the model were calculated from the likelihood ratio test (Tables IV and V). Apocrine stain result again added significantly to the prognostic value of the model. Other factors included in the model for survival were node status, menstrual status, tumour size and ER level, for both relapse free interval and survival. None of the other seven factors was found to be significant in this form of analysis for patients survival.

**Figure 1** Breast cancer sections ×200 stained with Zinc $\alpha_2$ Glycoprotein antibody showing (**a**) positive staining (**b**) equivocal and (**c**) negative staining.

**Table II** Breast carcinomas stained for zinc α-2 glycoprotein

| Type of carcinoma | Number of cases | Stain + | Stain ± | Stain − |
|---|---|---|---|---|
| Infiltrating ductal | 133 | 51 | 24 | 58 |
| Infiltrating lobular | 7 | 1 | 1 | 5 |
| Medullary | 2 | 0 | 0 | 2 |
| Mucoid | 2 | 0 | 2 | 0 |
| Papillary | 1 | 0 | 0 | 1 |
| Total | 145 | 52 | 27 | 66 |

**Table III** Relationship of prognostic factors to disease-free interval and survival by univariate analysis

| | Significance level DFI | Significance level Survival |
|---|---|---|
| Node status | 0.0001 | 0.0001 |
| Menstrual status | 0.0035 | 0.0001 |
| Tumour grade | 0.042 | 0.026 |
| Tumour size | 0.013 | 0.016 |
| ER level | 0.0029 | 0.005 |
| PgR status | 0.11 | 0.021 |
| Elastosis | 0.3 (NS) | 0.3 (NS) |
| Cellularity | 0.6 (NS) | 0.4 (NS) |
| Histology | 0.5 (NS) | 0.7 (NS) |
| Initial treatment | 0.2 (NS) | 0.2 (NS) |
| Adjuvant therapy | 0.3 (NS) | 0.4 (NS) |
| Apocrine stain | 0.03 | 0.03 |

NS, not significant. Tumour size was as a continuous variable. Stain was as present or absent.

### Discussion

Krompecher first described apocrine breast carcinoma in 1916 but difficulties in categorising apocrine change have made attempts at determining the clinical significance of such differentiation impossible.

The diagnosis of apocrine carcinoma has rested on the presence of large eosinophilic cells with basophilic nuclei and bulbous apical snouts seen on haematoxylin and eosin stained sections of breast cancer tissue (Azzopardi, 1979). The subjectiveness of this diagnosis is illustrated by the variety of claims of the incidence of apocrine change in breast cancer, ranging from 0.3 to 57%.

Haagensen (1986) has suggested that this sub-group of breast carcinomas may arise from areas of gross cystic disease with apocrine metaplasia. Zinc $\alpha_2$ glycoprotein forms 36% of the total protein content of apocrine sweat (Jirka, 1968) and is one of the major protein components of another apocrine secretion, breast cyst fluid (Haagensen *et al.*, 1979). A previous study (Bundred *et al.*, 1987*a*) has shown that staining of tumour cells with an antiserum to zinc $\alpha_2$ glycoprotein is a reliable immunohistochemical marker of apocrine differentiation in tumours. Workers using an antibody to another cyst protein, GCDFP 15, have confirmed that tumour apocrine differentiation can be objectively diagnosed using immunohistochemistry and found an incidence of 50–80% of tumours exhibiting some degree of apocrine change (Mazoujian *et al.*, 1983; Miller *et al.*, 1988; Le Dousal *et al.*, 1985). The incidence of 55% of tumours exhibiting staining in this study is in broad agreement with these figures.

Mossler *et al.* (1980), have claimed that apocrine carcinomas are deficient in oestrogen and progesterone receptors but we have been unable to find any relationship with either oestrogen or progesterone receptor status.

Two previous studies of tumour apocrine change using antisera to GCDFP 15 have shown an association between better differentiated tumours and immunohistochemically determined GCDFP 15 expression (Le Dousal *et al.*, 1985) and high cytosol GCDFP 15 levels (Silva *et al.*, 1982). We were able to substantiate these findings, the better differentiated carcinomas being less likely to be negative in comparision to the poorly differentiated tumours although the association was a weak one.

**Table IV** Relationship of prognostic factors to disease-free interval by multivariate analysis (145 cases)

| Factor | Groups (codes) | | Coeff. (β) | s.e (β) | P-value[a] |
|---|---|---|---|---|---|
| Node status | − ve (0) | | 1.433 | 0.283 | <0.0001 |
| | + ve (1) | | | | |
| Adjuvant therapy | none (A1 = 0, A2 = 0.) | (A1) | − 0.860 | 0.326 | 0.0001 |
| | tam (A1 = 1, A2 = 0.) | (A2) | − 1.630 | 0.457 | |
| | other (A1 = 0, A2 = 1.) | | | | |
| Tumour size | Not applicable | | 0.202 | 0.075 | 0.011 |
| ER level | <5 (0) | | − 0.672 | 0.264 | 0.014 |
| | ⩾5 (0) | | | | |
| Apocrine stain | 0% (0) | | 0.247 | 0.121 | 0.038 |
| | >0% (2) | | | | |
| Elastosis | 0 (E1 = 1, E2 = 0) | (E1) | 0.758 | 0.350 | 0.035 |
| | 1 (E1 = 1, E2 = 1) | (E2) | 0.031 | 0.297 | |
| | 2 (E1 = 0, E2 = 1) | | | | |

0 = none, 1 = minimal, 2 = marked. [a]Obtained from likelihood ratio test.

**Table V** Relationship of prognostic factors to survival by multivariate analysis (145 cases)

| Factor | Groups (codes) | Coeff. (β) | s.e. (β) | P-value[a] |
|---|---|---|---|---|
| Node status | − ve (0) | 1.058 | 0.289 | 0.0001 |
| | + ve (1) | | | |
| Menstrual status | pre (1) | − 1.322 | 0.386 | 0.0001 |
| | post (0) | | | |
| Tumour size | Not applicable | 0.265 | 0.081 | 0.0026 |
| ER level | <5 (0) | −0.926 | 0.274 | 0.0014 |
| | ⩾5 (1) | | | |
| Apocrine stain | 0% (0) | 0.263 | 0.135 | 0.047 |
| | >0% (2) | | | |

[a]Obtained from likelihood ratio test.

**Figure 2** Log rank curve of relapse-free interval (DFI) stratified by apocrine stain status ($P<0.05$).

**Figure 3** Log rank curve of survival in 145 breast cancers stratified by the presence or absence of apocrine staining: positively staining tumours have a poorer survival ($P<0.03$).

In many centres the only breast cancer tissue that is available will be formalin fixed and paraffin embedded. Various histological parameters can be assessed from haematoxylin and eosin stained sections of such material and some of these, e.g. histological grade, can be of prognostic significance (Elston *et al.*, 1982). However, a major criticism of these assessments is that they are subjective (Stenkvist *et al.*, 1979). The widespread use of immunohistochemistry has allowed the determination of more objective assessments of tumour behaviour. Immunohistochemical staining with the monoclonal antibody NCRC II, which recognises components of the milk fat globule membrane, can give prognostic information (Ellis *et al.*, 1985). However, the extent of staining relates to histological grade and oestrogen receptor status. Other prognostic markers which can be defined by immunohistochemistry such as epidermal growth factor receptor (Sainsbury *et al.*, 1987) require fresh tumour tissue for analysis. In this respect the immunohistochemical identification of apocrine change provides an independent prognostic parameter which does not rely on a complex staining scale assessment and can be applied to routinely fixed and processed tissue.

In many centres the trend towards conservative surgery for breast cancer (without determining node status) and the absence of oestrogen receptor assay facilities has meant that two of the most important prognostic factors are unavailable. Apocrine staining may prove a useful objective prognostic parameter in these patients and could be used to select patients who require adjuvant therapy. Furthermore, the recognition of apocrine differentiation in breast carcinomas as a clinicopathological entity with a distinct natural history may help therapeutically as such carcinomas are more likely to express androgen receptors (Miller, 1988) and less likely to respond to hormonal manipulation (Bundred *et al.*, 1987*b*).

Why apocrine differentiation should be associated with such a poor prognosis is not clear and must await further investigation. Despite the excess of women undergoing mastectomy and clearance whose tumours did not stain, the initial treatment made no difference to the prognosis of the carcinomas. This study shows that the identification of apocrine differentiation is of prognostic value and adds significantly to the predictive ability of the other standard prognostic factors.

We are grateful to Sir Patrick Forrest for allowing us to study patients under his care. The initial histological diagnoses were made by the Department of Pathology, University of Edinburgh. We thank Mrs W. Pitts for secretarial assistance.

### References

AZZOPARDI, J.G. (1979). *Problems in Breast Pathology*, p. 57, 341. W.B. Saunders: Philadelphia.

BONSER, G.H., DOSSETT, J.A. & JULL, J.W. (1961). *Human and Experimental Breast Cancer*. Pitman Medical: London.

BUNDRED, N.J., MILLER, W.R. & WALKER, R.A. (1987*a*). An immunohistochemical study of the tissue distribution of the breast cyst fluid protein, zinc $\alpha_2$ glycoprotein. *Histopathology*, **11**, 603.

BUNDRED, N.J., STEWART, H.J., STURGEON, C.M. & 4 others (1987*b*). Apocrine differentiation. Relationship to receptor status, prognosis and hormonal response in breast cancer. *Br. J. Surg.*, **74**, 1144.

ELLIS, I.O., HINTON, C.P., MACNAY, J. & 4 others (1985). Immunocytochemical staining of breast carcinoma with the monoclonal antibody. N.C.R.C. 11: a new prognostic indicator. *Br. Med. J.*, **290**, 881.

ELSTON, C.E., GRESHAM, G.A., RAO, G.S. & 4 others (1982). The Cancer Research Campaign (Kings/Cambridge) trial for early breast cancer: clinicopathological aspects. *Br. J. Cancer*, **45**, 655.

FISHER, E.R., GREGORIO, R.M. & FISHER, B. (1975). The pathology of invasive breast cancer. *Cancer*, **36**, 1.

HAAGENSEN, C.D. (1986). *Diseases of the Breast*, 3rd edn, p. 82, 250. W.B. Saunders: Philadelphia.

HAAGENSEN, D.E. Jr, MAZOUJIAN, G., DILLEY, W.G., PEDERSON, C.E., KISTER, S.J. & WELLS, S.A. Jr (1979). Breast gross cystic disease fluid analysis I, isolation and radioimmunoassay for a major component protein. *J. Natl Cancer Inst.*, **62**, 239.

HAWKINS, R.A., WHITE, G., BUNDRED, N.J. & 4 others (1987). Prognostic significance of oestrogen and progesterone receptor activities in breast cancer. *Br. J. Surg.*, **74**, 1009.

JIRKA, M. (1968). An alpha$_2$ globulin component present in sweat, saliva, tears, human milk, colostrum and cerumen. *FEBS Letts.*, **1**, 77.

KROMPECHER, E. (1916). Zur histogenese und morphologie der cystenmamma. *Bietz. Z. Path. Anat. U.Z. Al lg. Path.*, **63**, 403.

LE DOUSAL, V., ZANGERLE, P.F., COLLETTE, J. & 5 others (1985). Immunohistochemistry of a component protein of the breast cystic disease fluid with mol. wt. 15,000. *Eur. J. Cancer Clin. Oncol.*, **21**, 715.

MAZOUJIAN, G., PINKUS, G.S., DAVIS, S. & HAAGENSEN, D.E. Jr (1983). Immunohistochemistry of a gross cyst disease fluid protein (GCDFP 15) of the breast. *Am. J. Pathol.*, **110**, 105.

MILLER, W.R., SHIVAS, A.A., FRANCHIMONT, P. & HAAGENSEN, D.E. Jr (1988). Breast gross cystic disease protein 15 in human breast cancer in culture. *Eur. J. Cancer Clin. Oncol.*, **24**, 223.

MOSSLER, J.A., BARTON, T.K., BRINKHOUS, A.D., MCCARTY, K.S., MOYLAN, J.A. & MCCARTY, K.S. Jr (1980). Apocrine differentiation in human mammary carcinoma. *Cancer*, **46**, 2463.

SAINSBURY, J.R.C., FARNDON, J.R., NEEDHAM, G., MALCOLM, A.J. & HARRIS, A.L. (1987). Epidermal growth factor receptor status as predictor of early recurrence of and death from breast cancer. *Lancet*, **i**, 1398.

SILVA, J.S., COX, C.E., WELLS, S.A. Jr & 4 others (1982). Biochemical correlates of morphologic differentiation in human breast cancer. *Surgery*, **92**, 443.

STENKVIST, B.M., WESTMAN-NAESARS, S., VEGELIUS, J. & 4 others (1979). Analysis of reproducibility of subjective grading systems for breast carcinomas. *J. Clin. Pathol.*, **32**, 979.